# Mitochondrial Transcription Factor A (TFAM) Binds to RNA Containing 4-Way Junctions and Mitochondrial tRNA

**DOI:** 10.1371/journal.pone.0142436

**Published:** 2015-11-06

**Authors:** Timothy A. Brown, Ariana N. Tkachuk, David A. Clayton

**Affiliations:** Janelia Research Campus, Howard Hughes Medical Institute, Ashburn, Virginia, United States of America; Keio University, JAPAN

## Abstract

Mitochondrial DNA (mtDNA) is maintained within nucleoprotein complexes known as nucleoids. These structures are highly condensed by the DNA packaging protein, mitochondrial Transcription Factor A (TFAM). Nucleoids also include RNA, RNA:DNA hybrids, and are associated with proteins involved with RNA processing and mitochondrial ribosome biogenesis. Here we characterize the ability of TFAM to bind various RNA containing substrates in order to determine their role in TFAM distribution and function within the nucleoid. We find that TFAM binds to RNA-containing 4-way junctions but does not bind appreciably to RNA hairpins, internal loops, or linear RNA:DNA hybrids. Therefore the RNA within nucleoids largely excludes TFAM, and its distribution is not grossly altered with removal of RNA. Within the cell, TFAM binds to mitochondrial tRNAs, consistent with our RNA 4-way junction data. Kinetic binding assays and RNase-insensitive TFAM distribution indicate that DNA remains the preferred substrate within the nucleoid. However, TFAM binds to tRNA with nanomolar affinity and these complexes are not rare. TFAM-immunoprecipitated tRNAs have processed ends, suggesting that binding is not specific to RNA precursors. The amount of each immunoprecipitated tRNA is not well correlated with tRNA celluar abundance, indicating unequal TFAM binding preferences. TFAM-mt-tRNA interaction suggests potentially new functions for this protein.

## Introduction

Mitochondria are involved in numerous cellular functions, including many biosynthetic pathways, calcium signaling, apoptosis, innate immune responses, and production of ATP through oxidative phosphorylation (OXPHOS). Although most mitochondrial proteins are encoded in the nucleus and imported into the mitochondria, this organelle also contains its own genome. Mitochondrial DNA (mtDNA) encodes 13 proteins that are essential for OXPHOS, and also includes genes for two rRNAs and the 22 tRNAs that are needed for protein translation on mitochondrial ribosomes. The mtDNA exists in a nucleoprotein complex known as the mitochondrial nucleoid.

Mitochondrial Transcription Factor A (mt-TFA/ TFAM) is an HMG-box protein that was initially identified as a mtDNA transcription factor over 25 years ago in the Clayton lab [1]. Its role as a sequence-specific transcription factor at mitochondrial promoters has since been firmly established [2]. However, TFAM is an abundant protein with additional functions. The majority of TFAM is bound at sites dispersed over the full length of mtDNA where it bends mtDNA in maintaining nucleoid architecture [3,4]. We recently used TFAM as a proxy for determining the location and dimensions of the nucleoid using superresolution iPALM microscopy [5]. From those measurements we calculated that the mammalian nucleoid was packaged at a very high density. This was consistent with a model in which a compact core nucleoid structure would have to be selectively accessed by other transcription, replication, and repair proteins as needed. Mitochondrial RNA resides within close proximity to the nucleoid, and some of these transcripts co-purify with mtDNA in the form of RNA:DNA hybrids [6–8]. However, it is unclear if mitochondrial RNA plays a role in regulating the structure of the nucleoid or its access to specific proteins. We postulated that the abundance and close association of RNA with mtDNA had the potential to alter nucleoid structure via RNA:DNA hybrid formation, thus either facilitating or limiting protein access to the highly compact nucleoid core. In bacteria, the functional homologs of TFAM are the HU proteins, which are also DNA bending nucleoid architecture proteins that mediate various DNA transactions in prokaryotes. E. coli HU protein is thought to mediate enzymatic access to DNA in part by binding to complex nucleic acid structures such as branched DNA, RNA:DNA hybrids, and RNA [9,10]. One of our goals in this work was to characterize the ability of TFAM to bind to various RNA substrates toward defining the role of RNA within the TFAM-mediated architecture of the nucleoid.

In addition to RNA, mitochondrial nucleoids are also associated with numerous ribosomal proteins and other factors involved in protein synthesis [11,12]. TFAM was also found to be immunoprecipitated with the mitochondrial ribosomal recycling factor, mRRF [13]. Although most mitochondrial ribosomes are physically separated from the nucleoid, even a limited co-localization of nucleoid and ribosomal proteins implies that there is coordination at the level of biogenesis or function of these two nucleoprotein complexes. Through the course of characterizing TFAM interactions with RNA, we have discovered that TFAM is capable of binding RNA-containing 4-way junctions, and to mitochondrial tRNA, thus further expanding the potential roles of this protein in the mitochondria.

## Materials and Methods

### Preparation of TFAM protein and antisera

The mouse TFAM cDNA (IMAGE clone 3487433, RefSeqNM_009360.4) was amplified using the PCR primers 5’-agccatatgtccagcatgggcagctat-3’ and 5’-gctggatccttagtgttcggagatgtc-3’, and cloned into the NdeI and BamHI sites of pET28a (Novagen, EMD4 Biosciences). The resulting fusion protein excludes the N-terminal 32 amino acid mitochondrial localization sequence of TFAM, which is replaced with 23 amino acids containing a 6X HIS tag and the thrombin cleavage site. The clone was validated by nucleotide sequencing, and was used to transform the E. coli BL21(DE3) for expression using Overnight Express™ auto induction media (EMD4 Biosciences). Bacteria from 500 ml cultures were lysed using B-PER bacterial protein extraction reagent and purified using B-PER nickel-chelated columns (Thermo Scientific). In some experiments, the non-native 6X HIS leader was cleaved using 5 U of thrombin/mg of HIS-tagged TFAM. The cleaved peptide was removed by passage over nickel-chelated columns as above, and thrombin was removed using a HiTrap Benzamidine FF column (GE Healthcare). Purified protein was stored at -80°C in 50 mM Tris, 1 mM EDTA, 150 mM NaCl, 10% (v/v) glycerol.

Several commercially available antibodies against TFAM failed to perform in immunoprecipitation experiments. Therefore, a custom polyclonal antibody was generated in rabbits against bacterially expressed, HIS-tag purified, full-length mouse TFAM protein (Primm Biotech, Inc, Cambridge MA). The antibody was validated by Western blot analysis of cell lysates and by immunofluorescence imaging where the antibody stained mitochondrial nucleoids in a manner identical to an anti-DNA antibody (S1 Fig). Western analysis was done using 3T3Sw cell lysates from crosslinked cells as outlined below for RNA immunoprecipitation (RIP). Frozen cell pellets were lysed in RIP lysis buffer, treated with 0.03 U/μl of Benzonase® (EMD Millipore), and clarified via centrifugation at 9,400 x g for 10 min. Samples containing 30 μg protein were heated in reducing sample buffer at 75° C for 120 min prior to electrophoresis in a 4–12% PAGE gel in MES buffer. Western blots were incubated overnight with a 1:1000 dilution of TFAM antisera, followed by chemiluminescence detection with a 1:2000 dilution of Goat anti Rabbit IgG-HRP and LumiGlo substrate (Cell Signaling Technology). Imaging was done on a GE ImageQuant LAS400. Validation of the TFAM antibody by immunofluorescence is described below.

### Electrophoretic mobility shift assays

EMSA experiments were performed using the LightShift chemiluminescent EMSA system (Thermo Scientific/Pierce). Double-stranded nucleic acid substrates were generated by annealing equal volumes of complementary synthetic oligos at a concentration of 250 μM in 10 mM Tris-HCl pH 7.5. Annealing was performed by heating to 95°C for 5 min, cooled at a rate of 1°C/min for 70 min, followed by an additional 16–18 h at room temperature. Preparative 15% PAGE-TBE gels were used to separate single- from double-stranded nucleic acids, which were visualized with SYBR gold (Invitrogen) gel staining, followed by band excision, gel elution in 0.5 M sodium acetate, 10 mM Tris-HCl pH 7, 0.1 mM EDTA, and ethanol precipitation. Biotinylated EMSA substrate concentrations were determined by spectrophotometry (Nanodrop 2000) followed by chemiluminescence detection of membrane-immobilized substrates against known standards using an ImageQuant LAS400 Imager and ImageQuant TL (GE Healthcare). EMSA binding reactions for DNA substrates were done in 10 mM Tris HCl pH 7, 50 mM KCl, 1 mM DTT, 0.1 mg/ml BSA, 0.1% (w/v) NP40. For RNA substrates, 5 mM MgCl_2_ was also added. Reactions contained 20 fmoles of nucleic acid substrate and varying amounts of TFAM. Binding was allowed to occur for 30 min at 30°C prior to gel electrophoresis in 6% PAGE, 0.5X TBE gels (Invitrogen). Nucleic acids were transferred to positively charged nylon membrane (Roche), and crosslinked with UV light. Biotinylated substrates were detected using stabilized streptavidin-HRP/Luminol reagents as components of the Chemiluminescent Nucleic Acid Detection Module (Thermo Scientific). Quantification of free and bound nucleic acids was performed as above using the ImageQuant LAS400. Estimations of the apparent *K*_d_ values from EMSA data were determined from Hill plots where the x-intercept is the log (*K*_d_). Binding curves were plotted and linear regression analysis was done using GraphPad Prism. Several test substrates yielded two species of shifted bands, which likely represent multiple TFAM binding sites on these substrates [14]. We calculated the apparent *K*_d_ for these substrates from the combined TFAM-bound fraction. This value therefore does not reflect the *K*_d_ for either of the individual binding sites, but rather the overall binding of the substrate. TFAM was assayed for binding to a variety of DNA and RNA substrates generated as above using a series of oligonucleotides (Integrated DNA Technologies) as follows.

Light-strand promoter dsDNA; (5’-biotin-ATT TTT ACA AGT ACT AAA ATA TAA GTC ATA TTT TG-3’) and complementary oligo (5’-CAA AAT ATG ACT TAT ATT TTA GTA CTT GTA AAA AT-3’).

Randomized LSP dsDNA; (5’-biotin-ACT ACT GAA ATA CTT GAT TAT AGT ATT TAA TAT TT-3’) annealed to the complementary oligo (5’-AAA TAT TAA ATA CTA TAA TCA AGT ATT TCA GTA GT-3’).

Non-specific dsDNA 5681; (5’-biotin-AGA AGC AGG AGC AGG AAC AGG ATG AAC A-3’), annealed to its complementary DNA oligo. This sequence corresponds to mouse mtDNA sequence 5681–5708 with the CO1 gene.

Double-stranded RNA 5681; RNA oligo 5681 (5’-biotin-rArGrA rArGrC rArGrG rArGrC rArGrG rArArC rArGrG rArTrG rArArC rA-3’)annealed to its complementary RNA oligo (5’-rUrGrU rUrCrA rUrCrC rUrGrU rUrCrC rUrGrC rUrCrC rUrGrC rUrUrC rU-3’).

RNA:DNA hybrid substrates; RNA oligo LSP (5’-biotin-rArUrU rUrUrU rArCrA rArGrU rArCrU rArArA rArUrA rUrArA rGrUrC rArUrA rUrUrU rUrG-3’) annealed to the complementary LSP oligo above, and RNA oligo 5681 (above), annealed to its complementary DNA oligo (5’-TGT TCA TCC TGT TCC TGC TCC TGC TTC T-3’).

Single stranded RNAs; biotinlylated poly [rArC]_12_, and biotinylated poly [rU]_20_

Internal loop dsRNA substrates; RNA oligo (5’-biotin-rArGrU rArGrA rArGrC rArGrG rArArA rGrArA rGrUrG rArGrG rArUrG rArArC rArGrU rC-3’, annealed to 5’-rGrArC rUrGrU rUrCrA rUrCrC rUrGrU rGrCrC rArGrC rUrCrC rUrGrC rUrUrC rUrArC rU-3’, creating an 8 nt mismatch at the center of the substrate (underlined).

Stem-loop RNA substrate; RNA 5’-biotin-rArGrU rCrUrG rGrArC rArCrG rUrArC rUrUrU rUrUrG rUrArC rGrUrG rUrCrC rArGrA rCrU-3’, which self anneals, creating a 15 nt stem with a 5 nt bubble at the hairpin.

4-way junction oligos; DNA versions shown here. Corresponding RNA oligonucleotide set was also synthesized.

b strand, 5’-biotin-TCCGTCCTAGCAAGGGGCTGCTACCGGAAG-3’;

h strand, 5’-CTTCCGGTAGCAGCCTGAGCGGTGGTTGAA-3’;

r strand, 5’-TTCAACCACCGCTCAACTCAACTGCAGTCT-3’;

x strand, 5’-AGACTGCAGTTGAGTCCTTGCTAGGACGGA-3’

The FluoroTect™ GreenLys is a charged E. coli lysine tRNA labeled with a BODIPY fluorophore and was purchased from Promega. EMSA reactions with this substrate contained 316 fmoles of nucleic acid substrate and varying amounts of TFAM. Binding conditions were as described above. Imaging and analysis of gel shifted bands was done with an ImageQuant LAS400 Imager and ImageQuant TL (GE Healthcare) using the fluorescein LED and filter for the detection of BODIPY.

### Cell culture, immunofluorescence and imaging

GeneSwitch 3T3 cells (Invitrogen) were cultured in high glucose DMEM supplemented with 10% (v/v) newborn calf serum, 2 mM L-glutamine, 1 mM sodium pyruvate and [50 μg/ml] hygromycin-B (Invitrogen). For confocal microscopy, cells were grown in Lab-Tek II chambers with #1.5 borosilicate coverglass bottoms (Nunc) coated with 15 μg/ml human fibronectin (Millipore). Immunofluorescence was performed by fixing cells for 30 min at 37°C with 4% (w/v) paraformaldehyde in 0.1 M phosphate, pH7.4, permeabilized with 0.25% (w/v) triton X-100 in PBS. Where appropriate, samples were treated with either or both [0.1mg/ml] DNase-free RNase or [50 U/ml] RNase-free DNase (Roche) for 90 min at 37°C, blocked in 5% (v/v) goat serum and incubated with affinity purified anti-TFAM polyclonal antibody (Primm) diluted 1:3000 in 0.25% Triton X-100 in PBS overnight at 4°C. Cells were labeled with Alexa Fluor 488 goat anti-rabbit IgG secondary antibody (Invitrogen) diluted 1:4000 in 0.25% (w/v) Triton X-100 in PBS for 1 h at room temperature. DNA was labeled with anti-single-stranded DNA (anti-ssDNA) monoclonal antibody (clone BV16-19 [Millipore]) diluted 1:4000 as above, and secondarily with an Alexa Fluor 568 goat anti-mouse IgG secondary antibody (Invitrogen) diluted 1:3,000 as above.

Confocal imaging was performed on a Zeiss LSM 510 META microscope equipped with a 100x/1.4NA Plan-Apochromat objective, and the 488 line of a 30 mW multi-line gas argon laser. All images are 1024x1024, 12-bit z-stack acquisitions with 0.8 μm steps. Fluorescence per cell was quantified using Volocity (Perkin Elmer) to find objects by intensity and clipping objects to ROI. Microsoft Excel was used for statistical analysis.

### RNA immunoprecipitation (RIP)

Mouse 3T3Sw cells were grown in T-150 cm^2^ flasks to 80% confluency, washed with PBS and crosslinked with 1% (w/v) formaldehyde for 10 min. Crosslinking was quenched with excess glycine, and cells were again washed with PBS prior to harvesting by scraping and centrifugation. Cell pellets were frozen and stored at -80°C. Lysates were prepared by adding 0.6 ml of 50 mM Tris-HCl, pH 8.0, 1% (w/v) SDS, 10 mM EDTA, 50 U/ml SUPERase-In (Life Technologies), and protease inhibitors (cOmplete Ultra™, Roche) to each T-150cm^2^ derived pellet. Cell suspensions were sonicated in a Covaris TC13 tube using a Covaris S2 with a duty cycle of 5%, an intensity setting of 4, and 200 cycles per burst. This was run for seven repetitions of 2 min intervals. Lysates were diluted with 9 volumes of IP buffer [16.7 mM Tris-HCl pH 8.0, 167 mM NaCl, 1.2 mM EDTA, 1.1% (w/v) Triton X-100, 0.01% SDS (w/v), 50 U/ml SUPERase-In (Life Technologies), and protease inhibitors (cOmplete Ultra™, Roche)]. For each sample, 3 ml of lysate was treated with 6 μg of anti- TFAM polyclonal antibody (see above) and immune complexes were allowed to form at 4°C for 3 h with rotation. Immune complexes were immobilized by adding 50 μl of Dynabeads^®^ Protein G (Life Technologies), allowed to bind for 2 h at 4°C, and collected magnetically. Beads were sequentially washed once each with 1 ml of a low-salt buffer (20 mM Tris-HCl pH 8.0, 150 mM NaCl, 2 mM EDTA, 1% (w/v) Triton X-100, 0.1% (w/v) SDS), a high-salt buffer (20 mM Tris-HCl pH 8.0, 500 mM NaCl, 2 mM EDTA, 1% (w/v) Triton X-100, 0.1% (w/v) SDS), LiCl buffer (10 mM Tris-HCl pH 8.0, 250 mM LiCl, 1 mM EDTA, 1%(w/v) NP40, 1% (w/v) deoxycholate) and TE, pH 8.0. Immune complexes were then eluted with 100 mM sodium bicarbonate, 1% (w/v) SDS, 50 U/ml SUPERase-In. For RNA analysis, samples were treated with TURBO™ DNase (Life Technologies). Samples were then treated with proteinase K and crosslinks were reversed by adding 20 mM NaCl and heating at 65°C for 18 hr. Nucleic acids were precipitated in isopropanol, resuspended in water and stored at -20°C.

### Semi-quantitative RT-PCR and PCR

RNA samples were treated again with TURBO™ DNase (Life Technologies), followed by inactivation. cDNA synthesis was done using Superscript III RT (Life Technologies) as outlined by the manufacturer. Reverse transcriptase was omitted in parallel samples to control for contaminating DNA. The primers used for cDNA synthesis and amplification are listed in S1 Table. cDNA products were purified using MinElute columns (Qiagen), and either stored at -20°C or assayed immediately. Quantification of cDNA was done using a Lightcycler apparatus (Roche Applied Science) with standards generated from the plasmid p501-1, which contains the entire mouse mitochondrial genome, as outlined previously [15]. For quantification of mtDNA in the immunoprecipitations, the samples were assayed directly without DNase treatment or cDNA synthesis. Data reported are mean values of of 2–4 replicates with standard deviations typically being about 15% of the mean.

### Surface plasmon resonance (SPR)

TFAM binding kinetics to a dsDNA LSP sequence, a 4-way DNA junction, a 4-way RNA junction and to native mt tRNAs were obtained using a Biacore T100 (GE Healthcare Bio-Sciences) in a manner similar to that used previously [3]. Biotinylated nucleic acid substrates were immobilized at low densities on streptavidin (SA) series S sensor chips (GE Healthcare Bio-Sciences AB) to avoid mass transport limitations. Binding experiments were performed in triplicate by serial dilution of 6xHIS tagged TFAM in HBS-EP+ (10 mM HEPES pH. 7.4, 150 mM NaCl, 3 mM EDTA, and 0.05% (w/v) P-20) and injected at 50 μl/min (90 s contact, 360 s dissociation) over both reference and immobilized flow cell surfaces with an Rmax of 80–200 resonance units (RU). Regeneration between cycles was 0.1% (w/v) SDS at 10 μl/min for 15 s followed by a 60 s stabilization and HBS-EP+ buffer-only wash. All kinetics analyses (fit to a 1:1 binding model; parameters: local Rmax and RI constant = 0) were performed using Biacore T100 evaluation software 2.0.3 (GE Healthcare Bio-Sciences AB).

### Preparation of mitochondrial tRNAs

Mitochondrial tRNAs were prepared from mouse liver tissue as described [16]. Livers were isolated from mice after cervical dislocation under anesthesia in strict accordance with the recommendations in the Guide for the Care and Use of Laboratory Animals of the National Institutes of Health. The protocol (Number 13–101) was approved by the HHMI Janelia Research Campus Insitutional Animal Care and Use Committee. Briefly, isolated livers were homogenized in a Potter-Elvehjem homogenizer, and mitochondria were isolated by differential centrifugation. Mitochondria were treated with proteinase K and DNase followed by RNA extraction with phenol:chloroform: isoamyl alcohol. RNA was further purified by centrifugation in a 15–30% (w/v) sucrose gradient at 95,000 x g for 20h, isolating the small RNA fraction at the top of the gradient. tRNA was further isolated using a 5% PAGE/7 M urea gel in TBE. Gel-purified tRNA was excised from the gel and eluted for 6–8 h in 10 mM Tris-HCl pH 7.4, 150 mM NaCl, 1 mM EDTA and 0.2% (w/v) SDS. Transfer RNAs isolated under these nonacid conditions were presumed to be deacylated. Biotinylation of purified native mitochondrial tRNAs was accomplished using Pierce RNA 3’ End Biotinylation Kit per the manufacturer’s instructions with the following modifications; the initial denaturation step was omitted to preserve secondary structure and the ligation reaction was allowed to proceed overnight at 16°C (Thermo Fisher Scientific, Rockford, IL, USA).

### tRNA circularization and sequencing

TFAM -bound tRNA was isolated using RNA immunoprecipitation as outlined above. Total cellular RNA was isolated from cultured 3T3sw cells using the AllPrep DNA/RNA mini kit (Qiagen). RNA from each was circularized with T4 RNA ligase 1, followed by RT-PCR using the Superscript III first-strand synthesis kit (LifeTechnologies) per the manufacturers instructions. Primers tRNA V RT FS (5’-gtgtaggccagatgctttaata-3’) and tRNA V F (5’-ccagaagatttcatgaccaatg-3’) were used to amplify a 69-nt fragment, which was subcloned using the TOPO-TA cloning system (Life Technologies). Individual clones were isolated and the plasmids were sequenced using the M13 forward primer.

## Results

### TFAM does not bind simple RNA structures

**Fig 1:**
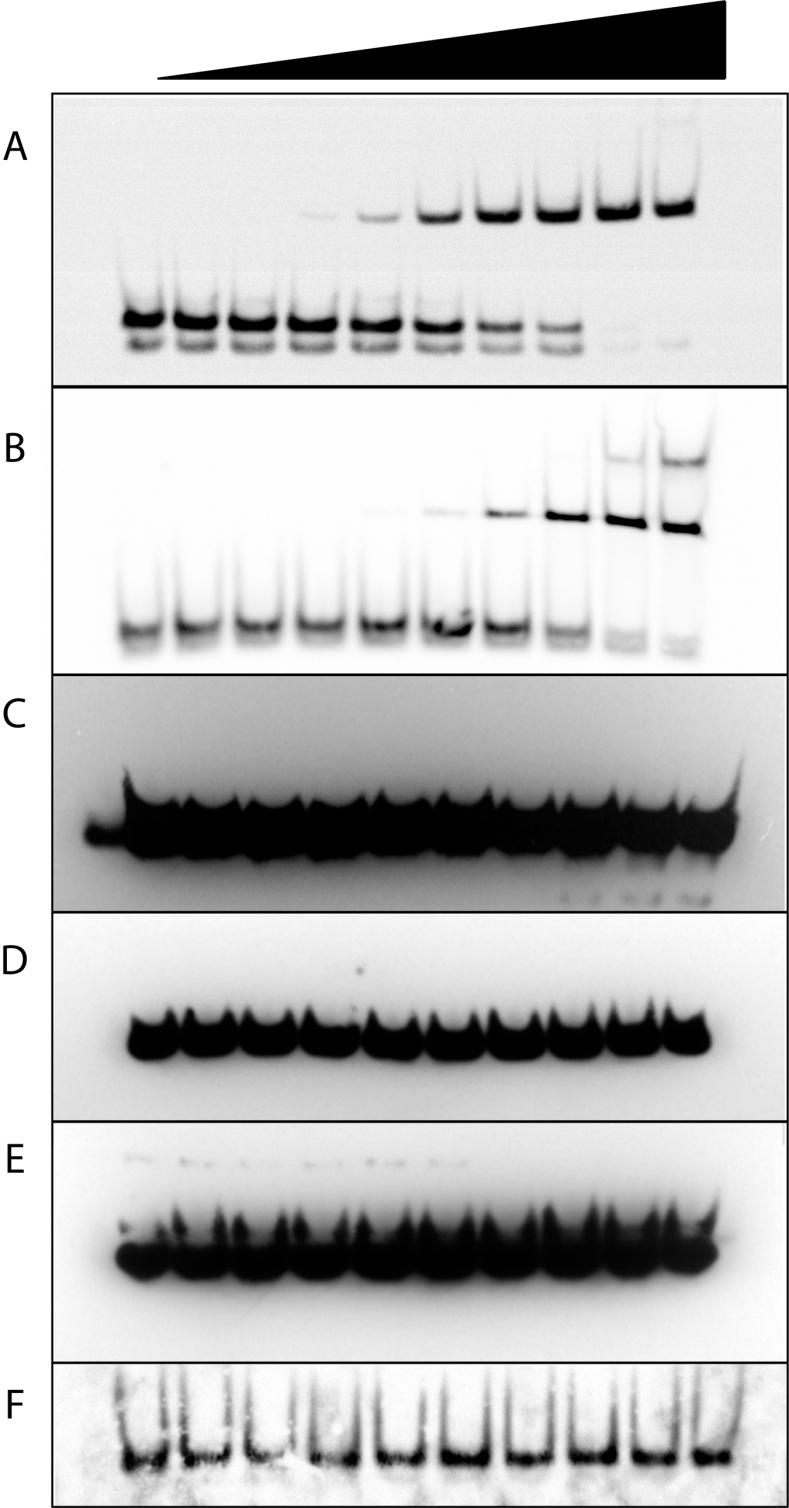
TFAM binding of linear DNA and RNA substrates by EMSA. Increasing amounts of TFAM were bound to 20 fM of each biotinylated substrate as follows; (A) dsDNA control sequence LSP, 0–1.44 μM TFAM with five-fold serial dilutions, apparent *K*_d_ of 6 nM, (B) dsDNA control sequence scrambled LSP, with serial dilutions of TFAM as in (A), apparent *K*_d_ of 100 nM, (C) dsRNA:DNA hybrid with 0–3.5 μM TFAM with two-fold serial dilutions, (D) ss poly [rArC]_12_, with TFAM dilutions as in panel (C), (E) poly [rU]_20_, with TFAM dilutions as in (C), (F) dsRNA^5681^, with TFAM dilutions as in panel (C).

The abundance of RNA in proximity with the nucleoid raises the possibility that various RNA structures might influence local DNA binding by TFAM. We first characterized TFAM binding of several linear nucleic acid structures using electrophoretic mobility shift assays (EMSA) (Fig 1). The mitochondrial light-strand promoter (LSP) double-stranded DNA (dsDNA) is the preferred binding site of TFAM [17,18] and is used here as a standard. In our EMSA system, purified TFAM binds the LSP dsDNA with an apparent *K*_d_ of 6 nM. This value is similar to that measured by others, thus validating the assay [18]. To measure non-specific TFAM binding to dsDNA, we randomized the LSP nucleotide sequence. We observe an apparent *K*_d_ of 100 nM for this substrate. With regard to RNA substrates, we demonstrate that TFAM is unable to bind to linear RNA:DNA hybrids, double-stranded RNA (dsRNA), or the single-stranded RNA (ssRNA) substrates poly (rArC) or poly(rU) (Fig 1C–1F)_._ Thus, these simple linear RNA structures are not bound by TFAM.

### TFAM binds to RNA internal loops and 4-way junctions containing RNA

**Fig 2:**
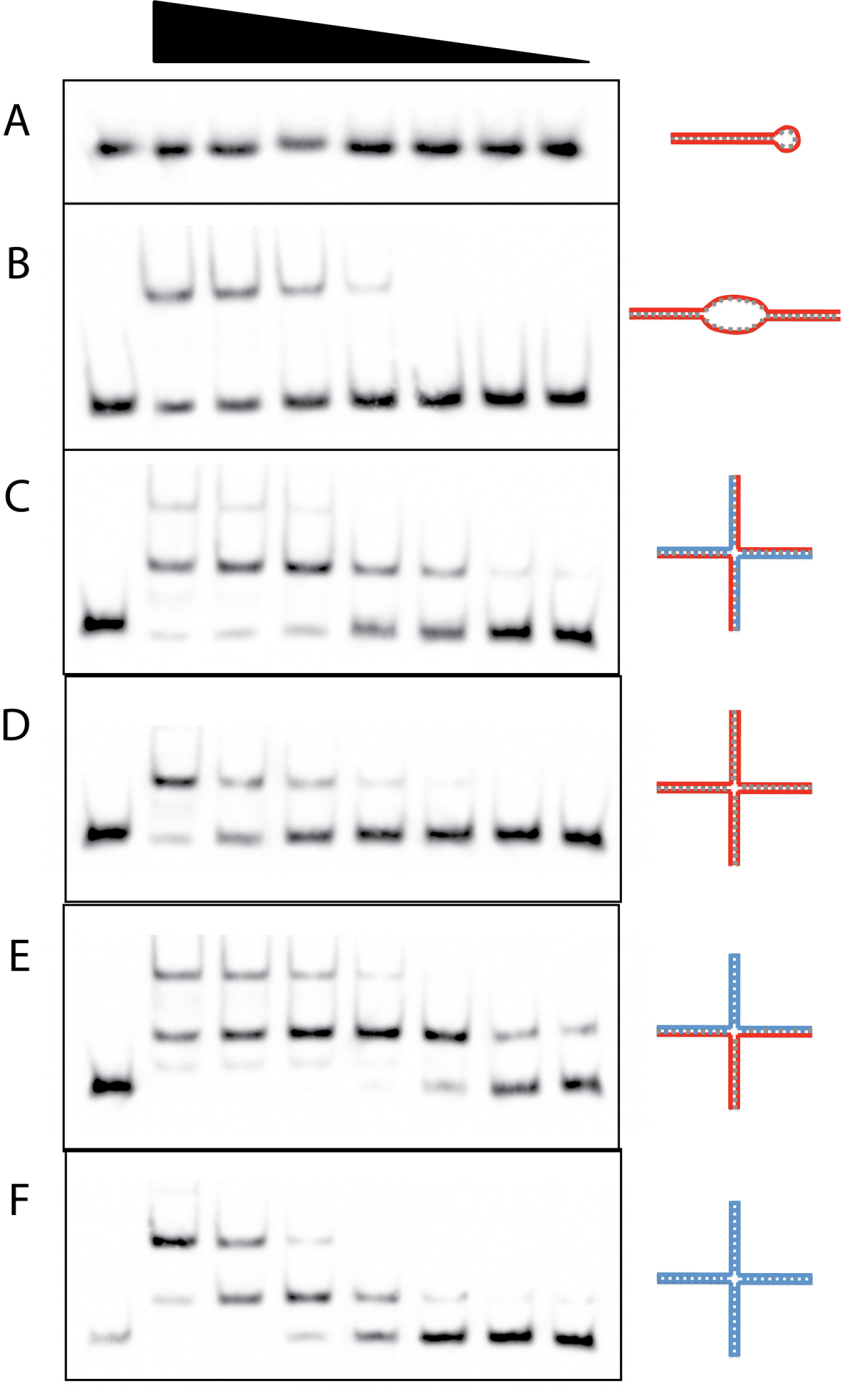
TFAM binding of complex DNA and RNA substrates by EMSA. Varying amounts of TFAM were bound to 20 fM of each biotinylated substrate with as follows; (A) Stem-loop RNA, 2.4–0.0375 μM TFAM with two-fold serial dilutions, (B) dsRNA with internal 8 nucleotide mismatch loop, TFAM dilutions as in (A), apparent *K*_d_ of 2.04 μM, (C) Alternating, four arm RNA:DNA 4-way junction, TFAM dilutions as in (A), apparent *K*_d_ of 299 nM, (D) RNA 4-way junction, 600–9.375 nM TFAM with two-fold serial dilutions, apparent *K*_d_ of 270 nM, (E) Mixed pairing RNA and DNA 4-way junction, TFAM dilutions as in (A), apparent *K*_d_ of 63 nM, (F) DNA 4-way junction, TFAM dilutions as in (D), apparent *K*_d_ of 63 nM. Left lane in each panel is free template without TFAM. Substrate diagrams appear to the right of each panel with RNA depicted in red and DNA in blue.

RNA most often forms structures that are not simply linear. We therefore assayed the capacity of TFAM to bind more complex substrates. EMSA data show that TFAM is unable to bind to a dsRNA hairpin loop, which is a common secondary structure in RNA (Fig 2A). However, TFAM is able to bind another common dsRNA structure that contains an internal loop (Fig 2B). This binding is weak and occurs with an apparent *K*_d_ of 2.04 μM, which is over 300-fold higher than the apparent *K*_d_ for the LSP dsDNA. TFAM has also been shown to bind to DNA 4-way junctions, which are typical of HMG-box proteins [14]. We have confirmed this and estimate TFAM binding with an apparent *K*_d_ of 63 nM. We then looked for the ability of TFAM to bind to RNA- and RNA:DNA-containing 4-way junction substrates and were able to qualitatively demonstrate binding. Several of the 4-way junction substrates yielded two binding isoforms that fill sequentially with increasing TFAM. The Hill coefficients for the 4-way substrates were 1.6–3, indicating cooperative binding of TFAM. This is consistent with previous studies in which TFAM binding to specific and nonspecific substrates yielded Hill coefficients of 1.5–2. [19]. We have estimated the equilibrium dissociation constants for these substrates by combining the bound forms and referring to the cumulative binding as the apparent *K*_d_. Binding affinity estimates indicate that the RNA-only 4-way junction has an apparent *K*_d_ of 270 nM (Fig 2D). The apparent affinity of TFAM for RNA:DNA hybrid 4-way junctions depended partially on the arrangement of the hybrid strands. The 4-way junction that is all RNA:DNA hybrid has an apparent *K*_d_ that is similar (~299 nM) to the all-RNA 4-way junction. However, when the 4-way structure contains some RNA:DNA, some RNA:RNA, and some DNA:DNA paired structure, the apparent TFAM affinity is greater (*K*_d_ ~63 nM), and is identical to the all-DNA 4-way junction (*K*_d_ ~63 nM). This likely indicates that TFAM is preferentially binding to the dsDNA arm of this substrate when given a mixed arrangement around the junction.

### RNA-immunoprecipitation reveals that TFAM binds to mitochondrial tRNA

**Fig 3:**
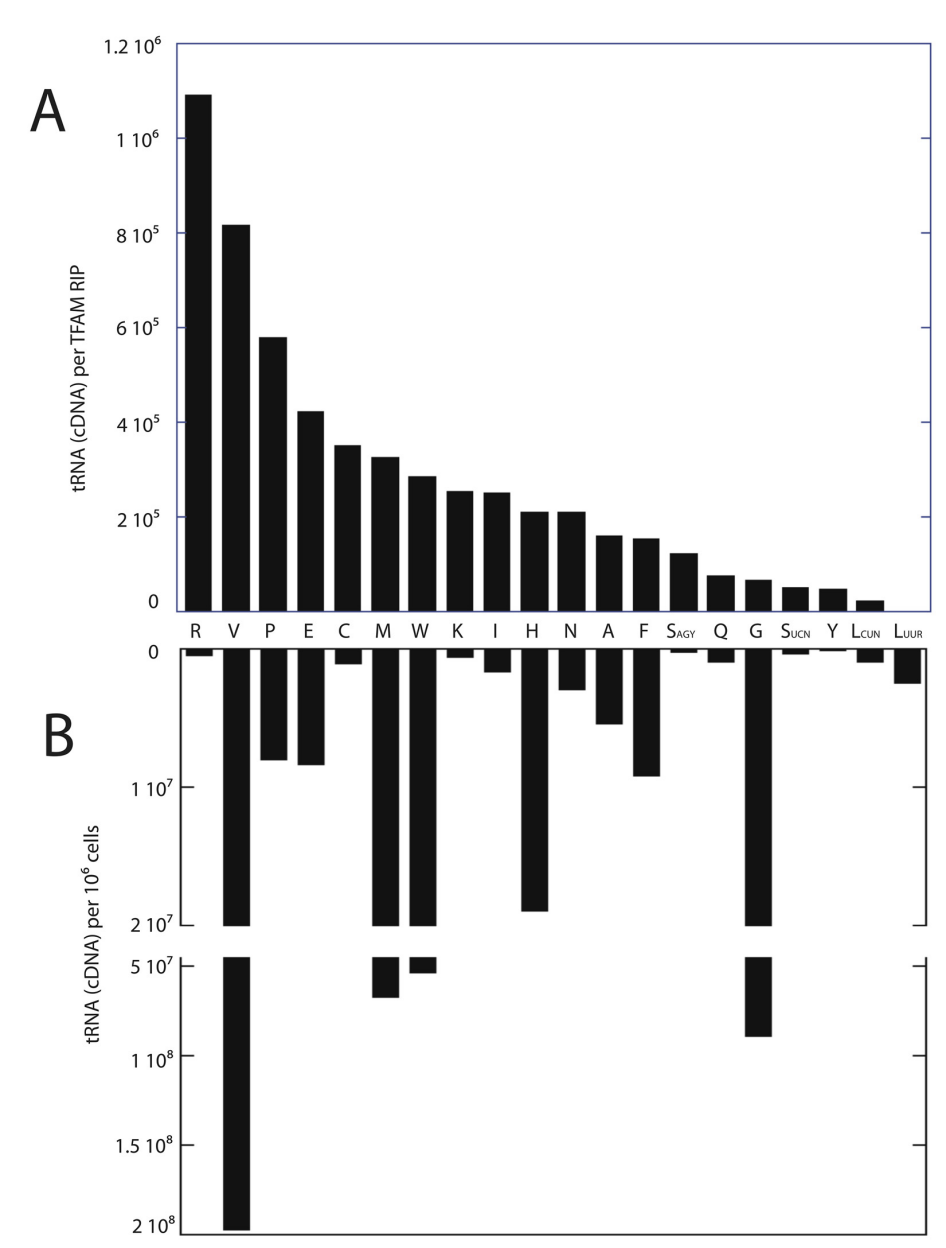
Relative levels of TFAM-immunoprecipitated and total cellular mitochondrial tRNAs determined by RT-PCR. (A) Ranked relative levels of mitochondrial tRNAs purified with TFAM in RNA immunoprecipitations. (B) Relative detectable levels of mitochondrial tRNAs obtained from 1x10^6^ 3T3sw cells.

Since the EMSA binding data indicated that TFAM binds to complex RNA structures, we sought to identify TFAM-bound mitochondrial RNAs by using RNA immunoprecipitation (RIP) [20]. We used quantitative RT-PCR to identify RNAs that co-purified with TFAM using formaldehyde cross-linked cells. Our data indicate that TFAM does not appreciably immunoprecipitate mitochondrial mRNAs. We failed to find RNAs corresponding to 12S rRNA, ND1, ND2, CO1, COII, COIII ATP8/6, COIII, ND3, ND4, ND5, ND6 and Cytb in either the sense or antisense direction. However, we were able to easily detect 20 of the 22 mitochondrial tRNAs. We were unable to design PCR primers suitable for the detection of mt-tRNA Asp and mt-tRNA Ile; therefore, these tRNAs are missing from our data set. A small amount of 16S rRNA was found in the immunoprecipitations at a level below that of all but mt-tRNA Leu^UUR^, which was below detection. The relative ability to detect tRNAs purified by TFAM RIP are ranked and displayed in Fig 3A. As shown, mitochondrial tRNA levels are detected over a broad range. We first sought to determine if this variation simply reflected the relative steady state levels of each tRNA within cells. We prepared total RNA from cells and performed quantitative RT-PCR to determine relative levels of mitochondrial tRNAs, and found little correlation between the amount of tRNA present in the cell and that which we purified by TFAM RIP (Fig 3B). The mt-tRNA Leu^UUR^ was undetectable in the TFAM immunoprecipitates, although our cellular assays indicated that this tRNA was more available than 9 of the other 19 tRNAs detected. Thus, although total cellular tRNA abundance may partially influence the amount that is bound to TFAM, it is not the primary factor. We also looked for TFAM binding correlations related to tRNA structural features without success. The determinants influencing the ability of TFAM to bind to a particular tRNA or our ability to detect those tRNAs is therefore not yet clear.

Transcription of the circular mtDNA proceeds from strand-specific promoters in opposite directions, creating both sense and antisense transcripts from both strands. We looked for the antisense RNAs corresponding to the tRNA regions and found that they were below detection in TFAM RIP samples. Therefore only bona fide tRNAs are detected in our TFAM immunoprecipitations. Finally, we assayed for cytoplasmic (non-mitochondrial) tRNA Arg, tRNA Pro, and tRNA Val to determine if TFAM was specifically binding mitochondrial tRNAs. We were unable to detect these cytoplasmic tRNAs in the TFAM RIP samples (data not shown), confirming our ability to cross link the TFAM to mitochondrial substrates in our immunoprecipitations.

### The majority of TFAM protein is bound to mtDNA *in vivo*

**Fig 4:**
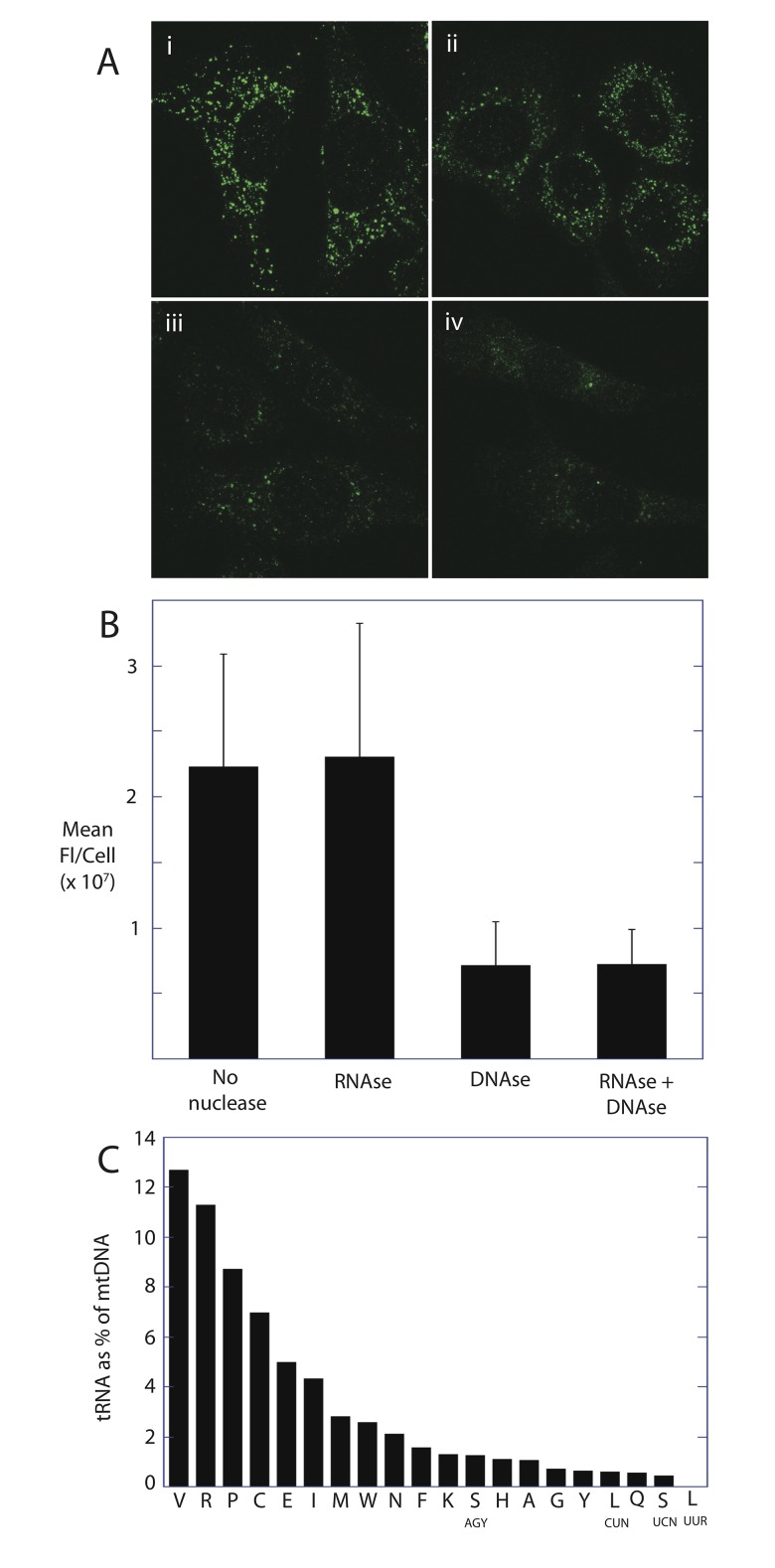
TFAM bound RNA represents a lesser fraction of TFAM bound DNA. (A) Immunofluorescence images of TFAM in untreated cells (i), cells treated with RNase (ii), DNase (iii) or both DNase and RNase (iv). (B) Relative mean fluorescence of TFAM retained during nuclease treatments shown in (A). (C) Levels of TFAM-bound tRNA relative to TFAM-bound to the corresponding mtDNA. Parallel RNA and DNA immunoprecipitations from the same samples were quantified by RT-PCR and PCR, respectively. Relative bound tRNA level is expressed as a percentage of bound mtDNA.

Given these in vitro and in vivo crosslinking data, only tRNA or RNA containing cloverleaf-like junctions would be expected to contribute to TFAM binding within the mitochondria or their nucleoids. We localized and quantified the relative amounts of DNA- and RNA-bound TFAM using cellular immunofluorescence. Fig 4A shows TFAM antibody immunofluorescence of mtDNA nucleoids. When cells are treated with RNase A, the cellular distribution of TFAM and mean fluorescence per cell is not significantly altered (Fig 4A ii and 4B). However, DNase treatment results in TFAM release and ablation of immunofluorescence. Further RNase treatment again did not alter the cellular distribution of TFAM or total fluorescence (Fig 4A iv and 4B). This indicates that RNA containing 4-way junction structures do not largely contribute to TFAM binding which largely occurs within the nucleoid. Fig 4C further shows that the relative levels of TFAM-bound tRNA represent a fraction of the TFAM bound to the corresponding mtDNA. We quantified the amount of tRNA and mtDNA in the parallel TFAM RNA and DNA-IP assays using RT-PCR and PCR, respectively. In this assay, the value obtained by the mtDNA PCR reflects the copy number of mtDNA. Site-specific primers used for both RT-PCR and PCR serve to normalize for slight differences in amplification efficiencies for each primer set. When the amount of tRNA detected is expressed as a percentage of the mtDNA (Fig 4C), it is apparent that tRNA represents a fraction of the TFAM-bound mtDNA. On average, the tRNAs represent only about 3% of the amount of mtDNA pulled down in parallel TFAM immunoprecipitations. This value however does not account for the loss of signal due to inefficiencies in first-strand DNA synthesis using reverse transcriptase in the RT-PCR. We tested the efficiency of reverse transcriptase using known amounts of tRNA transcripts generated from runoff transcription reactions, and found that the efficiency was 10–20%. The efficiency may in fact be lower with native tRNA which often have base modifications. Therefore, we conservatively estimate that the TFAM-bound tRNAs represent on average 15–30% of the amount of TFAM bound to the mtDNA. We might expect that this level of TFAM binding would be apparent in the RNase sensitive TFAM immunofluorescence (Fig 4A and 4B). The lack of this signal may be due to insensitivity to RNase, a more even distribution of the signal throughout the mitochondria, or loss of these complexes during cell fixation and permeabilization.

### Surface plasmon resonance reveals relative binding kinetics of TFAM to RNA and DNA substrates

**Fig 5:**
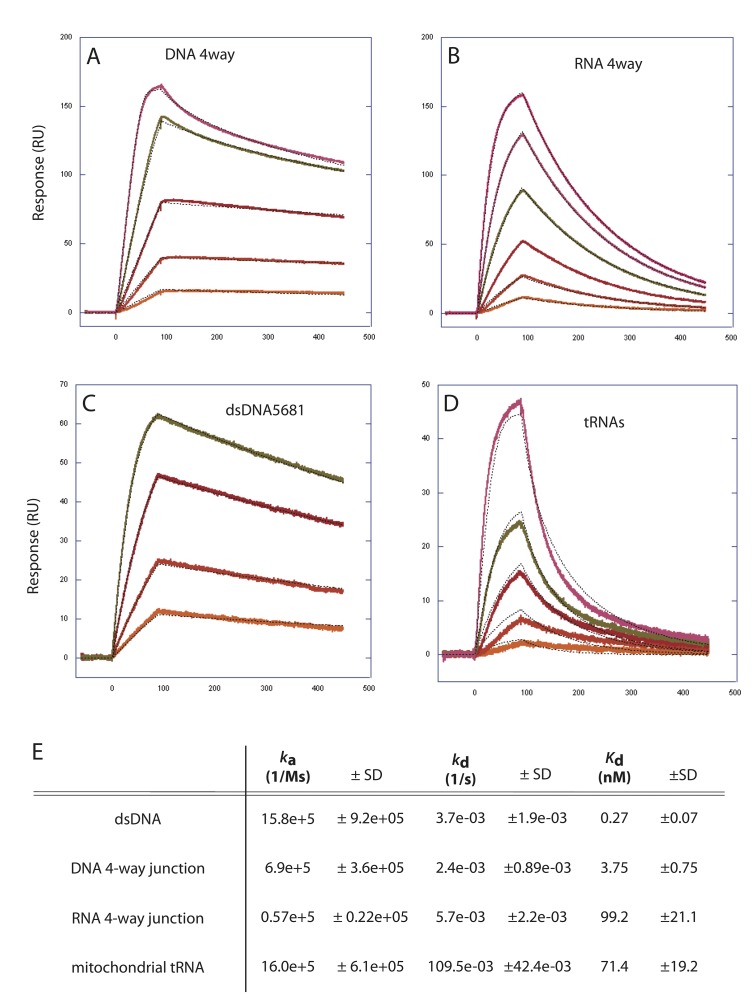
Binding kinetics of TFAM to RNA and DNA substrates using surface plasmon resonance. (A-D) Sensograms displaying the TFAM binding and dissociation rates of (A) a DNA 4-way junction, (B) an RNA 4-way junction, (C) linear double-stranded DNA, and (D) purified mitochondrial tRNAs. Individual tracings represent a single value in a range of TFAM concentrations in each of the experiments. (E) Kinetic data derived from these tracings include associate rate constant (*k*_a_), dissociate rate constant (*k*_d_) and the apparent dissociation constant (*K*_d_) for each of these substrates.

The EMSA derived binding affinities are complicated by multiple binding events (see Fig 2), imprecise curve fitting, and by the potential for in-gel dissociation of the protein-nucleic acid. To confirm apparent binding affinities and to obtain kinetic data, we used Surface Plasmon Resonance (SPR, Biacore). Fig 5 shows the SPR sensograms and binding kinetics of TFAM to a DNA 4-way junction, an RNA-4-way junction, dsDNA, and to purified mitochondrial tRNAs. TFAM has a very high affinity for all of these substrates. However, the affinities for the DNA substrates (*K*_d_ of 0.27 nM and 3.75 nM) are greater than those of the RNA substrates (*K*_d_ of 71 nM, 99 nM). The difference in these affinities is largely explained by the dissociate rate constants (*k*_d_). TFAM is more easily dissociated from the RNA substrates, and remains more steadily bound to the DNA substrates. Therefore, TFAM binding to the RNA 4-way junction and to mitochondrial tRNAs is more transient, relative to dsDNA and DNA 4-way junctions.

### TFAM-bound mitochondrial tRNAs are processed and have mature ends

**Fig 6:**
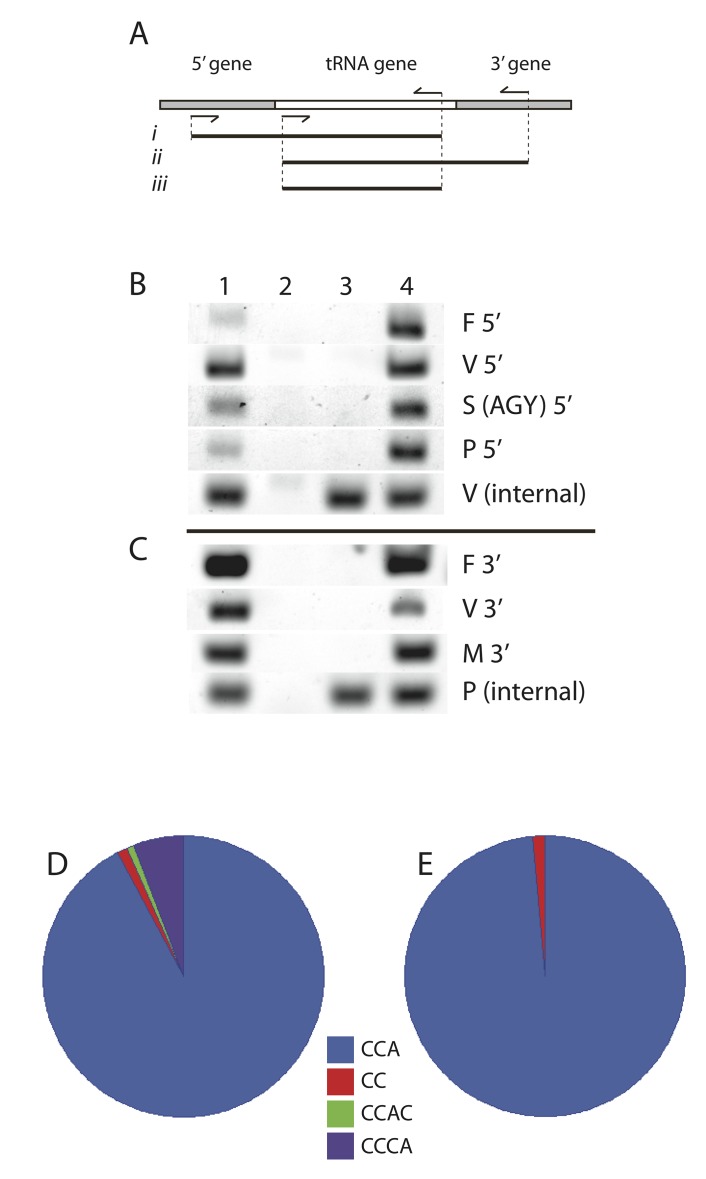
TFAM-bound mitochondrial tRNAs are processed and have mature ends. (A) Schematic for PCR detection of unprocessed tRNAs showing a tRNA flanked by putative RNA sequences from adjacent genes. PCR primer positions used on cDNAs are shown as arrows. Expected PCR fragments from unprocessed 5’ ends (i), unprocessed 3’ ends (ii) and internal tRNA (iii) are displayed. (B and C) PCR templates in lane 1 from total cellular cDNA, lane 2 templates made excluding reverse transcriptase, lane 3 templates are from TFAM-RNA IP, and lane 4 from TFAM-DNA IP. Samples for lanes 2, 3, and 4 are identical to those used for data obtained in Fig 4, which further controls for the sample preparation and PCR procedures. (B) PCR amplicons detecting 5’ flanking regions from each tRNA as in (Ai). V (internal) serves as a control for the TFAM-RIP reaction using tRNA internal primers as in (Aiii). (C) PCR amplicons detecting 3’ flanking regions from each tRNA as in (Aii). P(internal) serves as a control for RT-PCR using tRNA internal primers as in (Aiii). (D and E) 3’-end sequence frequency of multiple clones isolated from tRNA Val RNA circularization is pie-graph displayed. (D) tRNA Val sequences isolated from total cellular RNA, n = 104. (E) TFAM-RIP isolated tRNA Val sequences, n = 67.

Since mtDNA is packaged by TFAM, we postulated that it might bind tRNA transcripts immediately after transcription. TFAM binding of nascent RNA might indicate involvement in early steps of tRNA maturation. We used RT-PCR to determine if TFAM binds tRNAs that have yet to undergo end-processing by looking for longer versions of these RNAs. Fig 6 shows that TFAM immunoprecipitated mt-tRNA Phe, Val, Ser^AGY^, and Pro were all fully processed at their 5’-ends. Similarly, mt-tRNAs for Phe, Val, and Met were fully processed at their 3’-ends. All of these tRNAs were only detectable as processed versions, using primers within the boundaries of normal 5’- and 3’-ends.

We then employed RNA circularization [8] to determine the nucleotide sequence of the 5’- and 3’- ends of mt-tRNA Val from TFAM-RIP samples. In this assay, joining the ends with RNA ligase circularizes the immunoprecipitated tRNA. After DNA strand synthesis with reverse transcriptase, PCR is used to amplify the ligated region containing the joined ends. The resulting PCR fragment is cloned, isolated, and sequenced to reveal the tRNA ends at the nucleotide level. All mt-tRNA Val recovered by TFAM-RIP had the mature 5’-CATA-end. The vast majority of mt-tRNA Val also had a mature -CCA addition at the 3’ end (Fig 6E). Thus, TFAM binds to tRNA that has been modified by RNase P, RNase Z, and the CCA-adding enzyme [ATP (CTP):tRNA nucleotidyltransferase].

### TFAM is able to bind an aminoacylated tRNA

**Fig 7:**
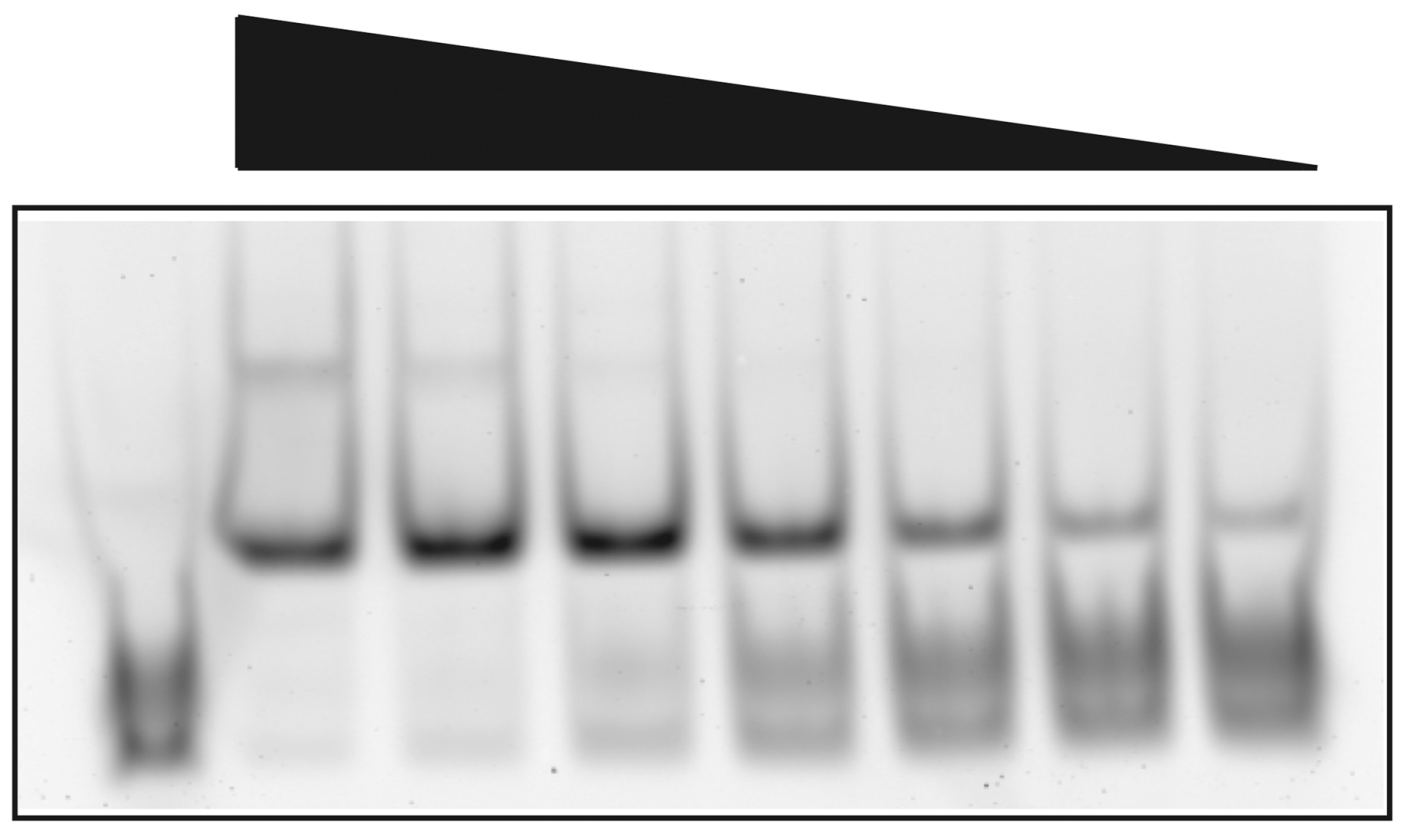
TFAM EMSA using FluoroTect™ GreenLys acylated tRNA. Varying amounts of TFAM were bound to 316 fM of BODIPY-labeled, acylated tRNA Lys from 1.48–0.093 μM TFAM using two-fold serial dilutions from left to right. Lane 1 (left) contains free tRNA template without TFAM. Measurement of bound and free substrates yields an estimated *K*_d_ of 20.4 nM.

Mitochondrial tRNAs which contain the 3’-CCA addition are mature substrates for aminoacylation. tRNAs that are charged with their appropriate amino acid are then presented to the ribosome during mitochondrial protein translation. We asked if TFAM is able to bind to an aminoacylated tRNA. To do this we employed a stable commercial tRNA Lys that carries a BODIPY-FL fluorescent tag (FluorotTect™ GreenLys). Fig 7 displays an EMSA showing that TFAM binds to the fluorescent tRNA Lys in a concentration dependent manner. Measurement of free and bound substrate yields an estimated *K*_d_ of 20.4 nM. Thus TFAM is capable of binding this aminoacylated tRNA with an affinity which is similar and perhaps even higher than what we measured for total mitochondrial tRNAs using SPR (Fig 5).

## Discussion

In this work, we have characterized the ability of TFAM to bind to various RNA containing substrates in order to elucidate the potential roles of this protein in both nucleoid structure and mitochondrial RNA function.

TFAM is the predominant architectural protein of the nucleoid, where it is involved in both transcription and maintenance of mtDNA. This HMG-box protein binds with high affinity to mtDNA promoters, bending and opening the DNA for initiation of transcription. Apart from the promoter regions, TFAM also binds to mtDNA nonspecifically; condensing it via DNA bending and loop formation [3,21]. The binding density of TFAM is likely to be a determinant of nucleoid structure and enzymatic function. The density has been estimated from ensemble averages and is controversial. By some measurements, TFAM density is very high in nucleoids, potentially binding mtDNA every ~17 base pairs [22]. However, excessive amounts of TFAM inhibit transcription in vitro [23] and overexpression of TFAM in mice decreases both transcription and replication [24–26]. It has been argued that TFAM levels have been overestimated, and that TFAM must be maintained at a level which is compatible with transcription [27]. The controversy highlights our lack of understanding regarding how TFAM binding events within mtDNA are modulated. Such events are likely to be dynamic and critically important in facilitating the accessibility of transcription, replication, and repair proteins. Disassembly of the nucleoid has recently been proposed to occur through TFAM phosphorylation, which inhibits TFAM binding and promotes its degradation by Lon protease [28,29]. TFAM also has the ability to slide extensively along DNA in vitro, and forms discrete and stable patches of protein through cooperative binding [30]. An alternative way to regulate TFAM binding might be through altering the local mtDNA topology. One of our goals was to test the feasibility that RNA might affect TFAM binding. We previously found that short RNA:DNA hybrids are prevalent throughout the length of closed circular mtDNA [8], but the influence of these hybrids on nucleoid structure was unknown. Data presented here indicate that TFAM does not bind to simple RNA:DNA hybrids in vitro. Thus, it is possible that simple linear RNA:DNA hybrids within mtDNA may constrain the ability of TFAM to bind or slide along native mtDNA, potentially influencing both the location and frequency of TFAM patch formation. We have also found that TFAM binds with high affinity to RNA hybridized to DNA within 4-way junctions. During replication of mtDNA, there is a large amount of single-stranded DNA that is displaced in the expanded D-loop that forms between the origins of replication. This ssDNA is often hybridized with RNA [31,32], creating a situation that is amenable to forming not only simple RNA:DNA hybrids, but also to RNA:DNA 4-way junction formation. Damas et al have found that common mtDNA deletions are defined by sites that are predicted to form such non-B DNA conformations in this region, and especially at tRNA loci [33]. However, the actual frequency and location of such RNA:DNA 4-way hybrids remains unknown. We show that the amount of TFAM bound to RNA in nucleoids is relatively small (Fig 4), indicating that if such structures exist, they are relatively sparse. However, TFAM binding to such structures may prove to be locally and incidentally important for enzymatic transactions.

We have also found that TFAM binds with nanomolar affinity to mitochondrial tRNAs. TFAM RNA immunoprecipitations did not yield mitochondrial mRNAs, antisense mitochondrial tRNAs, or non-mitochondrial tRNAs. This does not exclude the possibility that TFAM may bind some or all of these RNAs in vivo. Mitochondrial tRNAs are over 100-fold more abundant than mitochondrial mRNAs [34]. These and other potential substrates may simply fall below our ability to detect them. However, we present evidence that among RNA containing substrates, TFAM prefers those with cloverleaf-like junctions that are found in tRNAs. We have further characterized this interaction in order to narrow its functional implications.

Since TFAM is an abundant DNA binding protein, we first postulated that TFAM might bind to nascent RNA as it is displaced from mtDNA during transcription. Mammalian mitochondrial RNAs are transcribed as polycistronic RNAs originating from two strand-specific promoters on the circular mtDNA. Genes within mtDNA lack introns, and are most often arranged with tRNA genes interspersed between the mRNAs. The excision of tRNAs is coincident with rRNA and mRNA processing [35]. Thus, the first step of mitochondrial RNA maturation is the processing of tRNA by Ribonuclease P at the 5’-ends [36], and RNase Z at the 3’-ends of tRNAs [37]. We considered that TFAM might modulate this processing by binding to immature tRNA regions within polycistronic RNAs. However, we found that polycistronic RNAs do not co-purify with TFAM (Fig 6A). Furthermore, circularization of TFAM-bound mt-tRNA Val followed by RT-PCR demonstrates that it contains mature 5’- and 3’-ends. In addition, this tRNA has also gained the mature CCA-3’ sequence addition. This essential trinucleotide 3’-end terminus is not encoded by genome, but is added enzymatically to mature tRNAs [38]. This post-transcriptional modification is a prerequisite to the final step of tRNA biogenesis, which is the aminoacylation of each tRNA with its respective amino acid. Finally, we have demonstrated that TFAM is able to bind a fully mature, aminoacylated tRNA labeled with BODIPY. Although we cannot formally rule out a role of TFAM in tRNA processing, it appears that TFAM is not binding to nascent transcripts, but to mature tRNAs. During the course of this work, He et al also showed that a large amount of TFAM co-purifies with tRNAs in iodoxanol gradients [12]. We estimate that 15–30% of TFAM is bound to tRNAs, which appears to be congruous with those data. Although the authors do not comment on this co-sedimentation, our data substantiate this observation.

There have been numerous experimental links made between nucleoid proteins and their involvement in RNA processing, ribosome biogenesis, or translation. In both yeast and humans, the mitochondrial RNA polymerase amino terminal domain binds to proteins involved in RNA processing and translation [39–42]. Proteomic studies of purified nucleoids and immunoprecipitation of the nucleoid proteins TFAM and SSB have also identified many proteins involved in RNA processing and translation [11,12,43]. The translation elongation factor EF-Tu has been identified as an abundant nucleoid protein and as a TFAM interacting protein in immunoprecipitation experiments [12,44]. EF-Tu specifically binds to aminoacylated tRNAs (aa-tRNA) which presents the aa-tRNA to the ribosome during translation. We have measured the overall *K*_d_ of TFAM to a mixed population of mitochondrial tRNAs to be about 74 nM, which is equivalent to the equilibrium dissociation constant of bovine mitochondrial EF-Tu-GTP-Phe-tRNA (76 nM)[45]. The binding affinities are therefore at least consistent with the notion of a functional interaction or exchange of these proteins on tRNAs. In another series of experiments, nucleoid-proximal RNA granules have been identified that contain newly synthesized RNA and the RNA processing enzyme RNase P [46,47]. Bogenhagen has further demonstrated that RNA processing and early ribosome assembly also occurs in subcomplexes adjacent to nucleoids [48,49]. It has recently been discovered that mitochondrial tRNA Val is an integral structural component of the mitochondrial ribosome [50]. This tRNA is abundantly represented as a TFAM binding partner in our RIP assays, opening the possibility that TFAM may play a role in ribosome assembly via tRNA Val interaction. These associations suggest that there is coordination between transcription within the nucleoid and events leading to protein translation. Our discovery that TFAM interacts with mitochondrial tRNAs extends this implication.

Alternatively, TFAM may fill a more general need for a mitochondrial tRNA chaperone-like function. Recent evidence indicates that mitochondrial tRNA structure may require stabilization. The C-terminal region of mitochondrial leucyl-tRNA synthetase is able to bind and rescue mutant tRNAs, functioning as a tRNA chaperone which stabilizes the appropriate tRNA structure [51,52]. This chaperone function is independent of the holoenzyme activity and appears to be tRNA specific. Mitochondrial tRNAs in general are unique in that they are structurally relaxed [53]. Although this feature has been exploited for their purification [54], this instability has also hindered their study. Misfolded mitochondrial tRNAs have complicated the study of aminoacyl tRNA synthetases and EF-Tu binding, as only a fraction of purified tRNAs remain in their correct conformation in vitro [45].

Finally, mitochondria play a central role in both apoptosis and the innate immune response [55] and we cannot rule out a role for the TFAM-tRNA interaction in these processes. Mitochondrial tRNAs have been shown to regulate apoptosis by binding to cytochrome c [56]. TFAM is a member of the HMG-box family of proteins that have a remarkable range of functions [57]. The canonical HMGB1 protein has also been shown to bind branched RNAs [58]. Although the function of HMGB1 RNA binding is unclear, it has been considered to play a role in nucleic acid-activated innate immune responses [59]. Thus, in addition to being a nuclear DNA binding protein, HMGB1 is also a “danger signal” protein that mediates inflammatory responses when released from dying cells [60]. More recently, TFAM deficiency has been shown to elicit an antiviral innate immune response, caused by the escape of mitochondrial nucleic acid [61]. Speculation here on the role of TFAM in various mitochondrial tRNA-mediated processes hopefully will serve to promote further investigation into these possibilities. Regardless, the ability to bind mitochondrial tRNA expands the repertoire of TFAM, which also plays critical roles in mtDNA transcription and in maintenance of mtDNA architecture, establishing it as a pleiotropic mitochondrial nucleic acid facilitator.

## Supporting Information

S1 Fig Validation of rabbit antisera against mouse TFAM protein. (A) Confocal immunofluorescence image with TFAM antisera and AlexFluor 488 conjugated secondary antibody. (B) Anti-DNA antibody immunofluorescence with Alexa-Fluor 568 conjugated secondary antibody. (C) Merged images from (A) and (B) demonstrating co-localization of TFAM and DNA antisera at mtDNA nucleoids. Scale bar in (A) is 10 μm. (D) TFAM antisera Western analysis showing asterisk-labeled TFAM band at appropriate migration size, and lack of significant cross-reactivity.
(PDF)

S1 Table Oligonucleotide primers used in RT-PCR and PCR reactions. (DOCX)
