# Supplementary figures and images for: Mitochondrial Transcription Factor A (TFAM) Binds to RNA Containing 4-Way Junctions and Mitochondrial tRNA
Source: PLoS One. 2015 Nov 6;10(11):e0142436. doi: 10.1371/journal.pone.0142436 (PMC4636309; doi:10.1371/journal.pone.0142436)

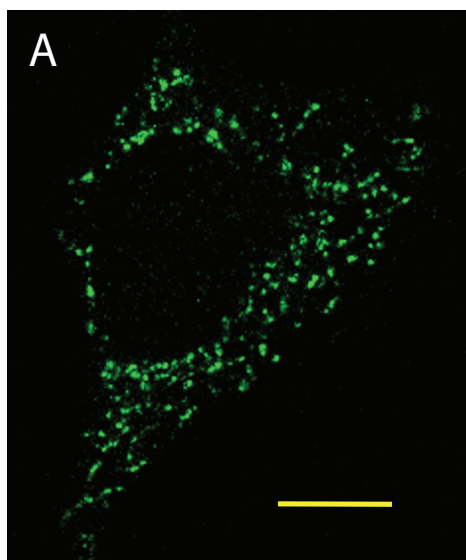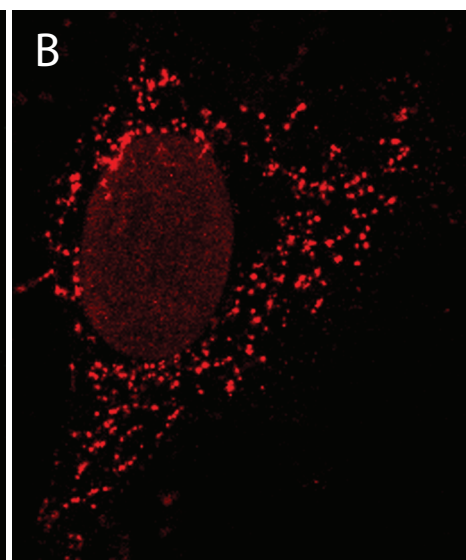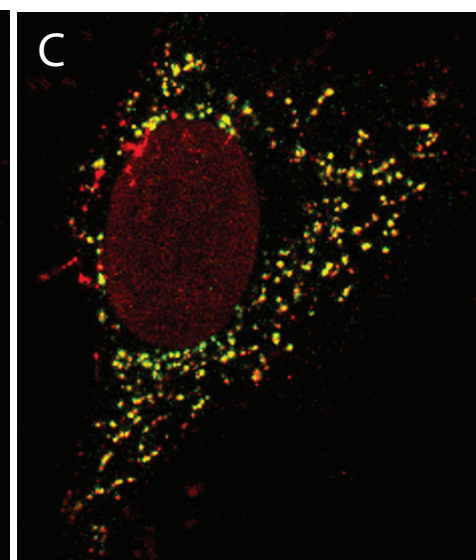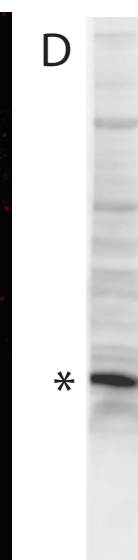

Supplement: S1 Fig — (A) Confocal immunofluorescence image with TFAM antisera and AlexFluor 488 conjugated secondary antibody. (B) Anti-DNA antibody immunofluorescence with Alexa-Fluor 568 conjugated secondary antibody. (C) Merged images from (A) and (B) demonstrating co-localization of TFAM and DNA antisera at mtDNA nucleoids. Scale bar in (A) is 10 μm. (D) TFAM antisera Western analysis showing asterisk-labeled TFAM band at appropriate migration size, and lack of significant cross-reactivity. (PDF) [file pone.0142436.s001.pdf]
