# Supplementary material for: Mitochondrial Transcription Factor A (TFAM) Binds to RNA Containing 4-Way Junctions and Mitochondrial tRNA
Source: PLoS One. 2015 Nov 6;10(11):e0142436. doi: 10.1371/journal.pone.0142436 (PMC4636309; doi:10.1371/journal.pone.0142436)
Supplement: S1 Table — (DOCX) [file pone.0142436.s002.docx]

| **Gene** | **strand** | | **Primer ID** | **Sequence** |
| --- | --- | --- | --- | --- |
| NCR | H | HSP fwdjunct | | tcaatgttccgtgaaccaaa |
| tRNA Phe | H | tRNA_F fwd | | aaagcaaagcactgaaaatgc |
|  | L | tRNA_F rev | | tgggatacaattatccatctaag |
| 12S rRNA | H | 12sf | | gccaatgaaatgggaagaaa |
|  | H | 12S bubble F | | gcaatgaagtacgcacacac |
|  | L | 12sr2fs | | gcttaccttgttacgacttatctcc |
|  | L | 12S revjunct | | caccggtctatggaggtttg |
| tRNA Val | H | mt trnavf | | ttaaagcatctggcctacacc |
|  | L | trnavr | | tcagagtgttcattggtcatg |
| 16S rRNA | H | 16S tail fwd | | gcaatccaggtcggtttcta |
|  | L | 16S tail rev | | cgcttatttgtaaggtggctct |
|  | L | 16S revjunct | | gtaggataaatgttttgattt |
|  | H | 16S fwdjunct | | cgtacaccctctaacctagagaagg |
| tRNALeu^UUR^ | H | tRNA L(UUR) NT | | cagagccaggaaattgcgta |
|  | L | tRNA L(UUR) rev | | gagccaggaaattgcgtaag |
| ND1 | H | ND1 fwd | | tccccattctaatcgccata |
|  | L | ND1 rev | | atgccgtatggaccaacaat |
| tRNA Ile | H | tRNA I fwd | | actttgatagagtaaattatagaggtt |
|  | L | tRNA I rev | | tagaaataagagggcttgaac |
| tRNA Gln | L | tRNA Q fwd | | aggtgcacggagaaattttg |
|  | H | tRNA Q rev | | ggacaataggaattgaacctacac |
| tRNA Met | H | tRNA M fwd | | ggcccataccccgaaaac |
|  | L | tRNA M rev | | acgggaaggatttaaaccaa |
| ND2 | H | ND2 fwd | | ttccaaccaacaataactcaaaaa |
|  | L | ND2 rev | | ggggctaggggtagggttat |
| tRNA Trp | H | mt trnawf | | tactagtccgcgagccttca |
|  | L | trnawr | | tgtgtgttttcttagggctttg |
| tRNA Ala | L | tRNA A fwd | | aagcaattgatttgcattc |
|  | H | tRNA A rev | | actgtaagacttcatcctacatct |
| tRNA Asn | L | tRNA N fwd | | tgaagccagtaatagggtatttagc |
|  | H | tRNA N rev | | tggcaggaattaaacctacg |
| tRNA Cys | L | tRNA C fwd | | ggtgatattcatgtcgaattg |
|  | H | tRNA C rev | | agagatttctctacaccttcg |
| tRNA Tyr | L | tRNA Y fwd | | ggtaaaatggctgagtaagca |
|  | H | tRNA Y rev | | ggatttaaacctctgtgtttagattt |
| CO1 | H | CO1 5’ LC | | gccccagatatagcattccc |
|  | L | CO1 3’ LC | | gttcatcctgttcctgctcc |
| tRNA Ser^UCN^ | L | tRNA S(UCN) fwd | | tggcttgaaaccaattttagg |
|  | H | tRNA S(UCN) rev | | aagaaaggaaggaatcgaacc |
| tRNA Asp | H | tRNA D fwd | | tagtaaaatcaattacataactttg |
|  | L | tRNA D rev | | tattgatctataatttaactttgac |
| COII | H | CO2 7200 forward | | tctcccctctctacgcattc |
|  | L | CO2 7200 FS | | gccctatggttttaacggt |
| tRNA Lys | H | tRNA K fwd | | aagagcgttaaccttttaagt |
|  | L | tRNA K rev | | tggagattttaaggtctctaacttt |
| ATPase8 | H | ATP8 fwd | | gccacaactagatacatcaacatga |
|  | L | ATP8 rev | | gtgccagtgggaatgtttgt |
| ATPase6 | H | ATP6 fwd | | cgcctaatcaacaaccgtct |
|  | L | ATP6 rev | | tcatgttcgtccttttggtg |
| COIII | H | COIII fwd | | ccgcagcatgatactgacat |
|  | L | COIII rev | | tcctcatcaataaatggagacgta |
| tRNA Gly | H | tRNA G fwd | | aactgacttccaattagtagattct |
|  | L | tRNA G rev | | actctcttctgggtttattcag |
| ND3 | H | ND3 fwd | | gcattctgactcccccaaat |
|  | L | ND3 rev | | gcagagcttgtagggtcgaa |
| tRNA Arg | H | tRNA R fwd | | aatgatttcgactcattagattat |
|  | L | tRNA R rev | | ttggtaattatgaacagcatca |
| ND4 | H | ND4 fwd | | caaccctcacacacacgaga |
|  | L | ND4 rev | | tcaggcctgtaattagttttgga |
| tRNA His | H | tRNA H fwd | | agactgtgaatctgacaacagga |
|  | L | tRNA H rev | | ggtgaataaggaggtttatttcc |
| tRNA Ser^AGY^ | H | tRNA S(AGY) fwd | | aagaaagattgcaagaactgct |
|  | L | tRNA S(AGY) rev | | atgtttttaaacatggaagcatg |
| tRNALeu^CUN^ | H | tRNA L (CUN) fwd | | ggtcttaggaaccaaaaacctt |
|  | L | tRNA L (CUN) rev | | acttttatttggatttgcacca |
| ND5 | H | ND5 11800 fwd | | ttcccactgtacaccaccac |
|  | L | ND5 11800 FS | | tatattctatattattgtgg |
|  | H | ND5 12200 fwd | | tgatggtacggacgaacaga |
|  | L | ND5 1200 FS | | tagggaaaatcaaactatagc |
| ND6 | L | ND6 fwd | | atgttggaaggagggattgg |
|  | H | ND6 rev | | tacccgcaaacaaagatcac |
| tRNA Glu | L | tRNA E fwd | | caacgatgatttttcatgtca |
|  | H | tRNA E rev | | agcattcaactgcgaccaat |
| Cytb | H | Cytb 14750 fwd | | tcaaacaacccaacaggatt |
|  | L | Cytb 14750 FS | | atgattaggatacctaggat |
| tRNA Thr | H | tRNA T fwd | | gtcttgatagtataaacattactctgg |
|  | L | tRNA T rev | | 5ttcatttcaggtttacaagacca |
| tRNA Pro | L | tRNA P fwd | | gaataccagctttgggtgct |
|  | H | tRNA P rev | | aaggagctactccccaccac |
| Cyt tRNA V | N/A | Cyto tRNA V fwd | | gtttccgtagtgtagtggttatc |
|  | N/A | Cyto tRNA V rev | | gtgtttccgcccggtttc |
| Cyt tRNA R | N/A | Cyto tRNA R fwd | | ccagtggcgcaatggataac |
|  | N/A | Cyto tRNA R rev | | gtcgaacctagaatcttctg |
| Cyt tRNA P | N/A | Cyto tRNA P fwd | | gctcgttggtctaggggtatg |
|  | N/A | Cyto tRNA P rev | | gctcgtccgggatttgaac |
|  |  |  | |  |
|  |  |  | |  |
